# A novel neck brace to characterize neck mobility impairments following neck dissection in head and neck cancer patients

**DOI:** 10.1017/wtc.2021.8

**Published:** 2021-07-12

**Authors:** Biing-Chwen Chang, Haohan Zhang, Sallie Long, Adetokunbo Obayemi, Scott H. Troob, Sunil K. Agrawal

**Affiliations:** 1 Department of Mechanical Engineering, School of Engineering and Applied Sciences, Columbia University, New York, New York, USA; 2 Department of Otolaryngology—Head and Neck Surgery, Columbia University Irving Medical Center, New York, New York, USA; 3 College of Physicians and Surgeons, Columbia University, New York, New York, USA; 4 New York Presbyterian-Columbia University Irving Medical Center, New York, New York, USA; 5 Department of Rehabilitative and Regenerative Medicine, College of Physicians and Surgeons, Columbia University, New York, New York, USA

**Keywords:** neck dissection, range of motion measurements, wearable neck brace

## Abstract

**Objective:**

This article introduces a dynamic neck brace to measure the full range of motion (RoM) of the head–neck. This easy-to-wear brace was used, along with surface electromyography (EMG), to study changes in movement characteristics after neck dissection (ND) in a clinical setting.

**Methods:**

The brace was inspired by the head–neck anatomy and was designed based on the head–neck movement of 10 healthy individuals. A 6 degrees-of-freedom open-chain structure was adopted to allow full RoM of the head–neck with respect to the shoulders. The physical model was realized by 3D printed materials and inexpensive sensors. Five subjects, who underwent unilateral selective ND, were assessed preoperative and postoperative using this prototype during the head–neck motions. Concurrent EMG measurements of their sternocleidomastoid, splenius capitis, and trapezius muscles were made.

**Results:**

Reduced RoM during lateral bending on both sides of the neck was observed after surgery, with a mean angle change of 8.03° on the dissected side (95% confidence intervals [CI], 3.11–12.94) and 9.29° on the nondissected side (95% CI, 4.88–13.69), where CI denotes the confidence interval. Axial rotation showed a reduction in the RoM by 5.37° (95% CI, 2.34–8.39) on the nondissection side. Neck extension showed a slight increase in the RoM by 3.15° (95% CI, 0.81–5.49) postoperatively.

**Conclusions:**

This brace may serve as a simple but useful tool in the clinic to document head–neck RoM changes in patients undergoing ND. Such a characterization may help clinicians evaluate the surgical procedure and guide the recovery of patients.

## Introduction

Head and neck cancers encompass a wide variety of cutaneous, salivary, and mucosal malignancies. These cancers often spread to the regional lymphatics of the neck, that is, the cervical lymph nodes (Shah et al., 2001). Neck dissection (ND) is a surgical method for removing cervical lymph nodes, suspected to contain deposits of cancer. ND requires manipulation of the 11th cranial nerve, that is, the spinal accessory nerve, which runs in an oblique line from the skull base to the back, innervating the neck sternocleidomastoid (SCM) and trapezius (TR) muscles. Injury to the 11th cranial nerve, may unfortunately, cause substantial morbidity post-surgery.

Cutaneous and mucosal cancers of the head and neck are the first and the sixth most common forms of cancer in the United States. These affect 5.4 million nonmelanoma skin cancer patients, 87,000 melanoma skin cancer patients, and 65,000 head and neck cancer patients per year (Howlader et al., 2017b). Denervation injury to the spinal accessory nerve occurs during ND (Dijkstra et al., 2001). Lack of innervation to the SCM and TR muscles results in pain and restricted range of motion (RoM) in the shoulders and neck, and significantly impacts patients’ quality of life (Eickmeyer et al., 2014). The confirmation of denervation is made by electromyography (EMG). During ND, manipulation of the spinal accessory nerve is required and this manipulation may result in transient or permanent injury to the spinal accessory nerve and muscle denervation of the SCM and TR muscles (Dijkstra et al., 2001). The lack of innervation of the SCM and TR muscles results in restricted RoM of the shoulder and neck and significantly impacts patients’ quality of life even years after the treatment (Erisen et al., 2004; Eickmeyer et al., 2014).

ND associated movement disorder is a clinical diagnosis. Exercise-based physiotherapy is often prescribed for the affected muscles as well as accessory muscles of the neck and shoulder girdle. Historically, the degree of movement restriction is measured by crude semi-quantitative assessment scales (Shah et al., 2001), protractors and goniometers (Erisen et al., 2004; Teymoortash et al., 2010), or a helmet with two inclinometers and a compass (Wilgen et al., 2004). Though patients perceive a benefit from therapy (Gallagher et al., 2015), there are currently few objective ways to guide the specific rehabilitative exercises or the duration of therapy (Lauchlan et al., 2008). Motion capture system and inertial measurement unit (IMU) are being used today as an angle measurement tool. Motion capture systems have high accuracy but require more time and ample space to set up and are less suitable to be used during patients’ routine clinical visits. IMUs are portable but usually suffer from the issue of signal drift due to the need to integrate velocity information to obtain position and orientation (Ahmad et al., 2013). Hence, a more reliable and portable measurement tool is needed for clinical assessment.

Inspired by the dynamic neck brace for the amyotrophic lateral sclerosis (ALS) patients (Zhang et al., 2019), we postulated that a neck brace could help clinicians observe the extent of the impairment and recovery trends over a period of time after surgery or during the follow-up physical therapy. The brace that was proposed for ALS patients uses a parallel mechanism and has 3 degrees-of-freedom (3-DOF) (Zhang and Agrawal, 2017). It allows only 70% of range of rotation of the head–neck. Thus, it cannot be used to measure the full RoM of the head–neck which may be critical for evaluating the changes in movement characteristics in patients undergoing ND procedures. The CarNeck is a hybrid cable-driven system that allows 3-DOF with full RoM (Shoaib et al., 2019). However, the 3-DOF design still interferes with the 6-DOF motion of the head. Another study proposed a 6-DOF parallel mechanism but is too bulky to transport between clinics (Wu et al., 2016). Hence, the objective of this article is to develop a wearable neck brace with 6-DOF that can characterize the full RoM of the head–neck. Furthermore, we propose a procedure that pairs the measurement of head–neck motion with measurement of neck muscle activity using surface EMG to characterize the head–neck movements of patients at the musculoskeletal level.

In the pilot study, this neck brace was used on five patients who underwent unilateral ND for cutaneous, oral cavity, or oropharyngeal squamous cell carcinoma. The neck kinematics and muscle EMGs during single-plane motions (sagittal plane flexion–extension, frontal plane lateral bending, and transverse plane axial rotation) were measured both before the surgery and 1 month postoperatively. These data show that this dynamic brace was capable of capturing the changes in movement characteristics of these subjects during their routine clinical visits with the surgeon.

## Methods

### Head–Neck Brace Design

**Figure 1.**
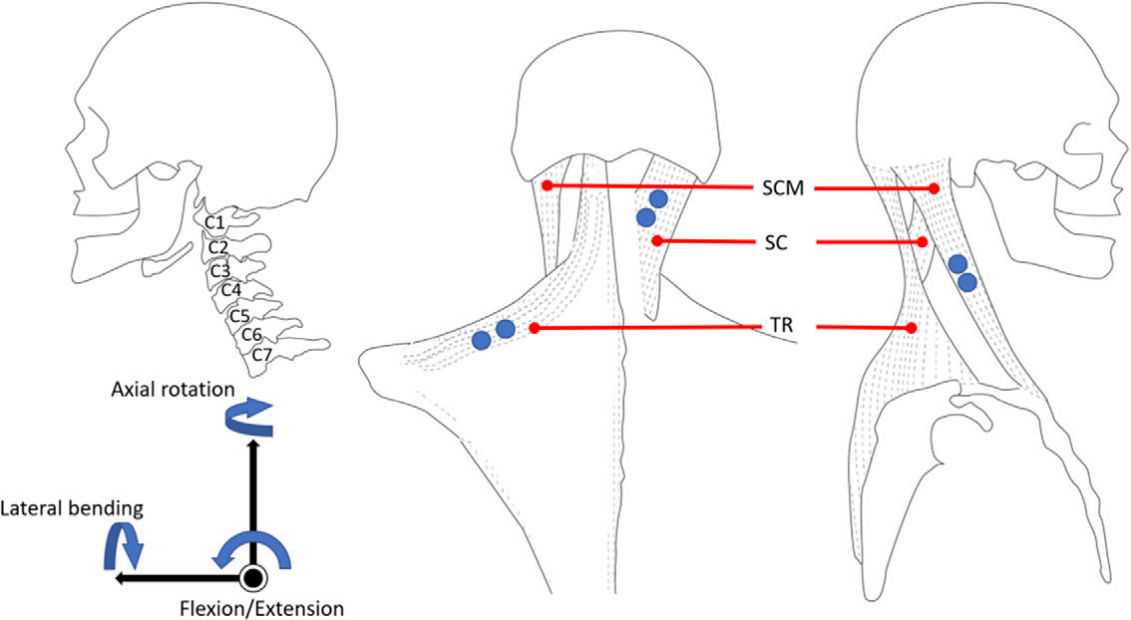
The anatomy and muscle location of the neck. (Left) A side view of the cervical vertebrae. (Middle and Right) The back view and side view of the muscle locations. Blue dots are the electromyography (EMG) electrode placements.

Previous studies show that both translation and orientation are involved in natural head–neck motion (Zhang and Agrawal, 2017). According to studies of the normal kinematics of cervical spine, a major contributor to the motion of the head is the atlanto-axial joint (i.e., the joint between the first and second vertebrae C1–C2, see Figure 1), along with limited motions of the other joints in the upper cervical spine (Lind et al., 1989; Bogduk and Mercer, 2000). Also, the flexion–extension of the head is contributed by the entire cervical vertebral column. In a previous study, the fabricated robotic neck brace allows users to rotate the head in all three axes coupled with small translations (Zhang and Agrawal, 2017). This parallel mechanism design of the neck brace has the advantages of high stiffness and low inertia, but occupies more space around the head due to its specific parallel linkage structure.

**Figure 2.**
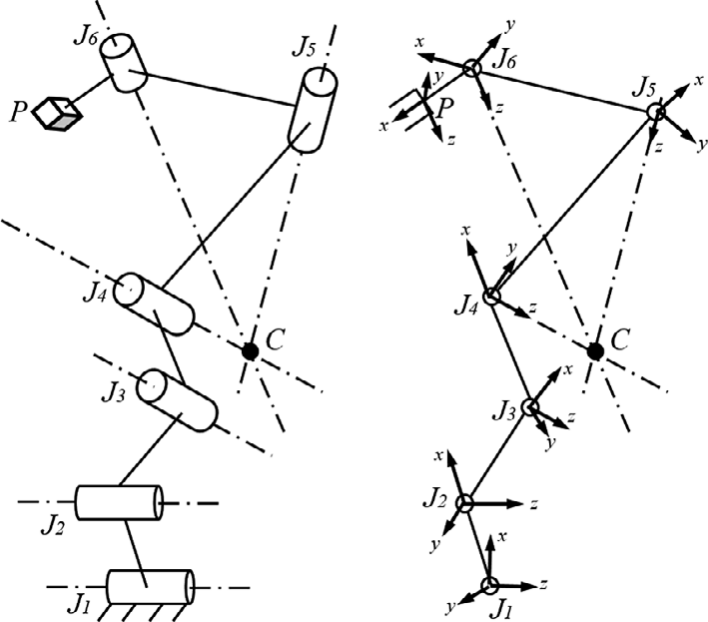
Schematic of the six revolute joint manipulator. The base joint is labeled *J*_1_ and the following joints are labeled *J*_2_, *J*_3_, *J*_4_, *J*_5_, and *J*_6_, respectively, in the sequence. The joint axes of the last three joints intersect at a point labeled *C* and the end-effector is labeled as *P.*

In the current design proposed in this article, the features of “light-weight” and “easy-to-use” are important design goals. We chose the structure of this neck brace as a six revolute-jointed serial chain mechanism. As shown in Figure 2, the structure is inspired by the anatomy (Figure 1) and the biomechanics of the neck motion (Lind et al., 1989; Bogduk and Mercer, 2000). The top cervical vertebrae (C1–C2) perform most of the head orientation, while the lower cervical vertebrae facilitate flexion–extension and lateral bending of the neck to change the Euclidean distance between C1 and C7. Hence, the motion can be decoupled into rotation at C1–C2 and translation from C2 to C7. In the design of the wearable robot, the displacement of the end-effector (P) is contributed by the three proximal joints *J*_1_,.., *J*_3_ and rotation from the three distal joints *J*_4_,.., *J*_6_. The lower cervical spine (C4–C7) provides the RoM for flexion–extension motion of the head–neck. Hence, the rotation axes of joints *J*_1_ and *J*_2_ were chosen parallel to the axis of flexion–extension of the head–neck in the design. To allow for lateral bending during flexion–extension, the axes of *J*_3_ and *J*_4_ are chosen perpendicular to *J*_1_ and *J*_2_. The axial rotation occurs mainly at the atlanto-axial joint with coupled motion in the other two planes. Therefore, we choose the axes of *J*_4_, *J*_5_, and *J*_6_ to intersect at a point *C* which anatomically aligns with the center of atlanto-axial joint to mimic a ball joint. Since the axial rotation of the head–neck is roughly around an axis that is geometrically along the cervical spine, *J*_6_ is placed distally along the head to align its joint axis along the cervical spine. The motion of the end-effector is governed by the Denavit–Hartenberg (D–H) model parameters such as the link lengths and joint offsets, as presented in Table 1.

**Table 1.** D–H parameters of the six revolute joint manipulator

Link	θ_i_ (rad)	*d_i_* (cm)	α_i_ (rad)	*a_i_* (cm)
1	*θ*_1_ ^a^	0	0	15
2	*θ*_2_ ^a^	0	π/2	10
3	*θ*_3_ ^a^	−16	0	5
4	*θ*_4_ ^a^	0	π/3	0
5	*θ*_5_ ^a^	0	π/3	0
6	*θ*_6_ ^a^	17	0	1.5

a Input variables.

The design goal of this device is to measure the full RoM of the head. During the design phase, the motion data of the head–neck over the full rotational range were recorded from 10 healthy individuals (26.8[4.26] years, range 24–29), using an eight-camera motion capture system (Vicon, Oxford, UK) with a sampling rate of 200 Hz. Eight retro-reflective markers were placed on the human body to form two rigid bodies. Four markers were placed on the trunk. One of these four markers was placed approximately on the cervical vertebrae (C7), where the base would be mounted. Other three markers were placed at front chest, and right and left acromion. A headband with four markers was placed on the head. One marker was placed approximately at the top of the atlas joint (C1). When designing the physical model, this marker helped locate the head’s position so that the linkage structure avoids colliding with the head. Another marker was placed on the top of the head, representing the end-effector position. Other two markers were at the left and right of the head. A static trial and four dynamic trials were recorded in this experiment. In the static trial, the upright neutral configuration of the subject was recorded. This posture was then used to construct a reference coordinate frame to describe the ensuing dynamic motions. In the dynamic trials, the subject was instructed to perform rotations within each of the three anatomical planes, followed by a spatial rolling movement of the head–neck that involved motions in all three planes. Each motion was repeated five times at self-selected speeds by the subject.

**Figure 3.**
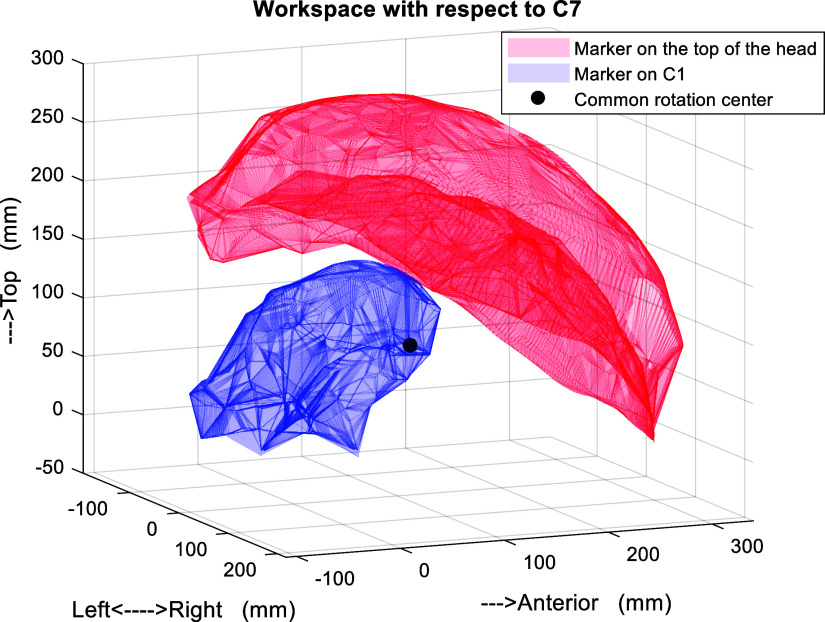
End-effector workspace from 10 subjects. The red space is the workspace created from the marker at the top of the head which the end-effector should reach. The blue space is created from the marker at C1, which the device should prevent from hitting. The black dot is the common rotation center of the red space. The point clouds are in the trunk coordinate for which the marker on C7 is the origin.

The data recorded from the subjects are visually displayed in Figure 3. The blue workspace shows the marker position C1 while the red workspace is for the reference point on the head. Since most of the head orientation happens at C1–C2, we assumed that the point cloud in the red workspace has a rotation center. A geometric approach was used to fit a sphere within the point cloud along with a center and a radius. These results helped us design the arc of linkages that connect the top three distal joints. We then chose the D–H model parameters of the neck brace through an exhaustive search so that the end-effector could reach all points within the workspace. To avoid the intermediate joints from hitting the head, the location of *J*_4_ was examined. A plane including the three markers at C1 and the shoulders was used to divide the workspace into a region for the head and for C1. *J*_4_ was chosen to be in region for C1. Additionally, the operation ranges of joint angles were defined such that they do not interfere with the head and neck during the dynamic movements.

**Figure 4.**
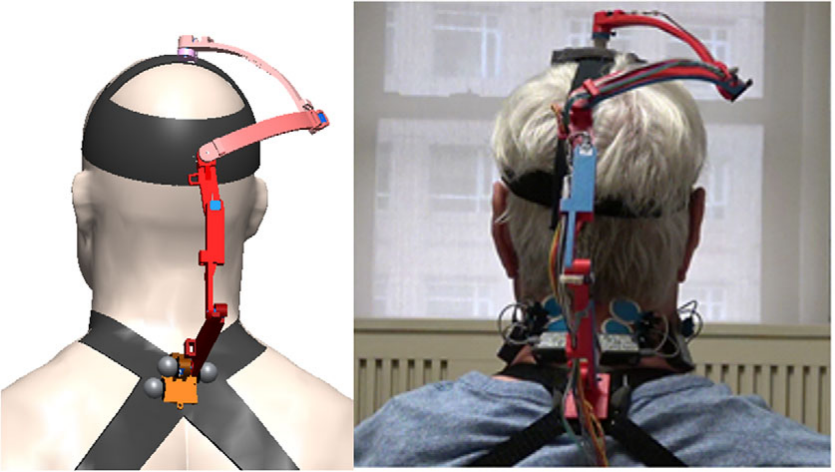
Schematic of the measurement brace and a picture of a subject using the brace. (Left) A CAD drawing of the measurement neck brace that consists of a series-chain with 6 degrees-of-freedom, designed for patients undergoing neck dissection for head and neck cancer. The base of the brace attaches to a rigid support worn by the user roughly around C7 vertebral segment. The end-effector of the series-chain attaches to a wearable cap. (Right) A participant wearing the brace during experimentation while sitting comfortably on a chair. Surface electrodes are mounted in the head and neck area to record muscle activity from bilateral sternocleidomastoid (SCM), splenius capitis (SC), and trapezius (TR) muscles.

The computer-aided design model and the actual device are shown in Figure 4. The base of the brace is on C7 and attaches to the human body through a pair of bands at the shoulders. A small bubble level is mounted on the base of the device to help position it relative to the shoulders. The end-effecter is attached to the top of the head with a soft elastic band. The linkages were 3D printed. The total weight of the brace, including the electronics, is 200 g which is about 1/25 of the weight of the human head. Each joint has a potentiometer along its rotational axis.

**Figure 5.**
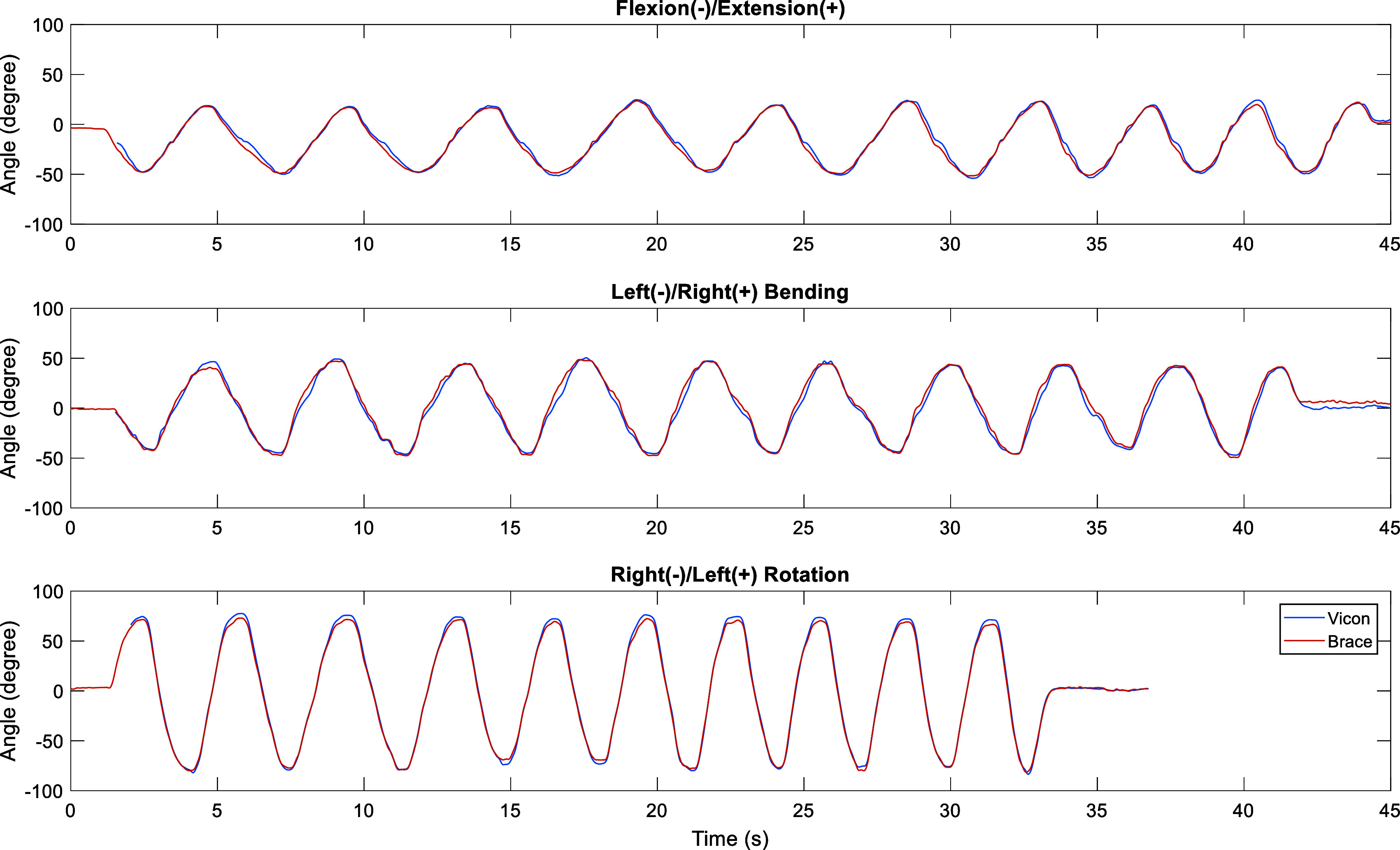
The comparison between motion capture system (blue line) and the brace (red line) from a representative subject performing three single-plane head orientations. Only primary head rotation is shown for each single-plane movement.

To address model mismatch between the theory and constructed brace in the presence of manufacturing and assembly tolerances, we took data from two subjects with the motion capture system when they performed head movements with the brace attached. Inverse kinematics was performed with the theoretical robot model given data from motion capture to find joint angles and simultaneously the potentiometer voltages were recorded at each joint. The potentiometer voltages were then polynomial fitted to the joint angles using the *fit* function in MATLAB to minimize errors between the head position/orientation computed by using the forward kinematics and the camera data. Once the model fitting was done, we validate the model with data from two additional subjects who were asked to do similar experiment as those by subjects for model matching experiment. The accuracy of the brace model was tested against the motion capture system. The performance in three single-plane motions from a representative subject is presented in Figure 5, and the group data is shown in Table 2. Maximum angle error was obtained from the maximum angle difference between the motion capture system and the brace for the group. Root mean square (RMS) error was computed with the mean RMS error over trials and over subjects. The maximum angle errors are below 6° and the RMS errors are below 5° in single-plane motion.

**Table 2.** Angular errors from validation data

Angle error (°)	Flexion–extension	Lateral bending	Axial rotation	Rolling
Maximum	5.73	5.90	5.44	8.17
RMS(SD)	4.10(0.97)	3.93(1.87)	4.06(0.77)	6.91(1.84)

Abbreviations: RMS, root mean square; SD, standard deviation.

### Experiments with Cancer Patients

Five patients, without prior surgery or radiation, who underwent unilateral selective ND for cutaneous, oral cavity, or oropharyngeal squamous cell carcinoma were evaluated preoperatively and 4 weeks postoperatively. Four weeks was chosen as the optimal time point when denervation injury would be evident, while the impact of postoperative swelling or incisional pain would be minimal. Also, this time point allowed for measurement prior to the initiation of any potential radiation therapy to avoid any confounding results. The mean(SD) age was 59(7.25) years (range 51–68 years). Clinical data regarding primary tumor type, extent of ND and postoperative data are summarized in Table 3. Selective lymphadenectomy was performed by the same surgeon on the deep jugular chain lymph nodes contained within selective regions I–V (as indicated by the primary tumor), which surround the spinal accessory nerve. In each case, the fascia connecting the spinal accessory nerve to the adjacent lymphatic and fibro-fatty tissue was atraumatically released and the nerve gently skeletonized and mobilized. There were no cases where the nerve was transected.

**Table 3.** Subject characteristics who participated in the experiment

	Mean(*SD*)	Range	S1	S2	S3	S4	S5
Age (years)	59 (7.25)	51–68	54	65	57	68	51
Height (cm)	177.8 (9.26)	150–177.8	188	185	165	173	178
Weight (kg)	86.4 (9.61)	54.4–80	98	91	75	78	90
Dissected side	–	–	Right	Right	Left	Right	Right
Neck levels dissected	–	–	1–4	2–4	Parotid, 2–4	1–4	1–4
Tumor primary			Oral cavity	Oropharynx	Cutaneous	Oral cavity	Oral cavity
Pathology			SCC	SCC	SCC	SCC	SCC
Adjuvant treatment			RT	CRT	CRT	–	–
Subjective shoulder dysfunction			No	No	Yes	No	No

Abbreviations: CRT, chemoradiation; RT, radiotherapy; SCC, squamous cell carcinoma.

The EMG electrodes were placed at six neck muscle sites, the SCM, the splenius capitis (SC), and the TR on both sides of the neck. The neck brace was then attached to the participant while they were asked to sit in an upright position on a chair. This determined the neutral configurations of the brace. The EMG signals were recorded wirelessly using TeleMyo DTS (Noraxon, AZ, USA). A microcontroller (NI myRIO-1900) was used to send a digital trigger between the neck brace and the EMG system for synchronization. These data were recorded and saved in the microcontroller with an interface on a laptop to visualize the data.

All participants sat comfortably on a chair during the measurements. The participants were first asked to keep their head in an upright neutral position, followed by three single-plane motion cycles with their head and neck—axial rotation in the transverse plane, lateral bending in the coronal plane, and flexion/extension in the sagittal plane. Each cycle started from the neutral position and ended at the neutral position. Each cycle had three sub-blocks: (a) Movement from the neutral to one extreme, (b) Movement from the first extreme to the second extreme, and (c) Movement from the second extreme to the neutral. The subjects performed each single-plane motion five times continuously at self-selected speeds. The experiment was done during the scheduled clinical visits of the subjects and it took about 30 min to complete the experiment.

The neck brace was sampled at 100 Hz and the EMG system was sampled at 1.5 kHz. The joint angles were low-pass filtered (zero lag fourth order Butterworth) at 6 Hz to reduce noise. The processing of the EMG signal from each channel was followed by: (a) filter the noise from cardiac beating using EMG software myoRESEARCH 3.10 (Noraxon, AZ, USA), (b) remove the DC offsets by subtracting the mean of each signal, (c) band-pass filter the data between 60 and 200 Hz, (d) full-wave rectify the signals, (e) create an envelope using moving averages with a window size of 300 data points (0.2 s), and (f) normalize the signal of each channel by the largest value recorded in that channel during all measurements of each participant in each visit. The continuous motion of each subject during a trial was segmented into five movement cycles. These cycles were averaged to compute the outcome variables. The cycles were segmented based on the primary angle of the motion, for example, flexion–extension during sagittal plane. They were then normalized with respect to time.

Data analysis was performed using MATLAB (The MathWorks Inc., Natick, MA, USA). Confidence intervals (95% confidence intervals [CI]) and paired *t* test was used to compare the mean values before surgery and 1 month postoperatively. The outcome variables include the completion time of each cycle, the changes in RoM in three anatomical planes, the maximum/minimum head angles in those planes, and the timing of the EMG peaks of each neck muscle before and 1-month after the surgical procedure of each subject. The statistical significance was set at *p <* .05.

## Results

In order to intuitively understand the single-plane motions of the head and neck, one can visualize the head as being connected to the shoulders by six ropes. The attachment points of these six ropes on the head and the shoulders are determined from the anatomical attachment points of the SCM, SC, and TR muscles in the human head and neck. A subset of these six ropes actuate, in coordination, to achieve the single-plane axial rotation, lateral bending, and flexion–extension of the head and neck.

**Figure 6.**
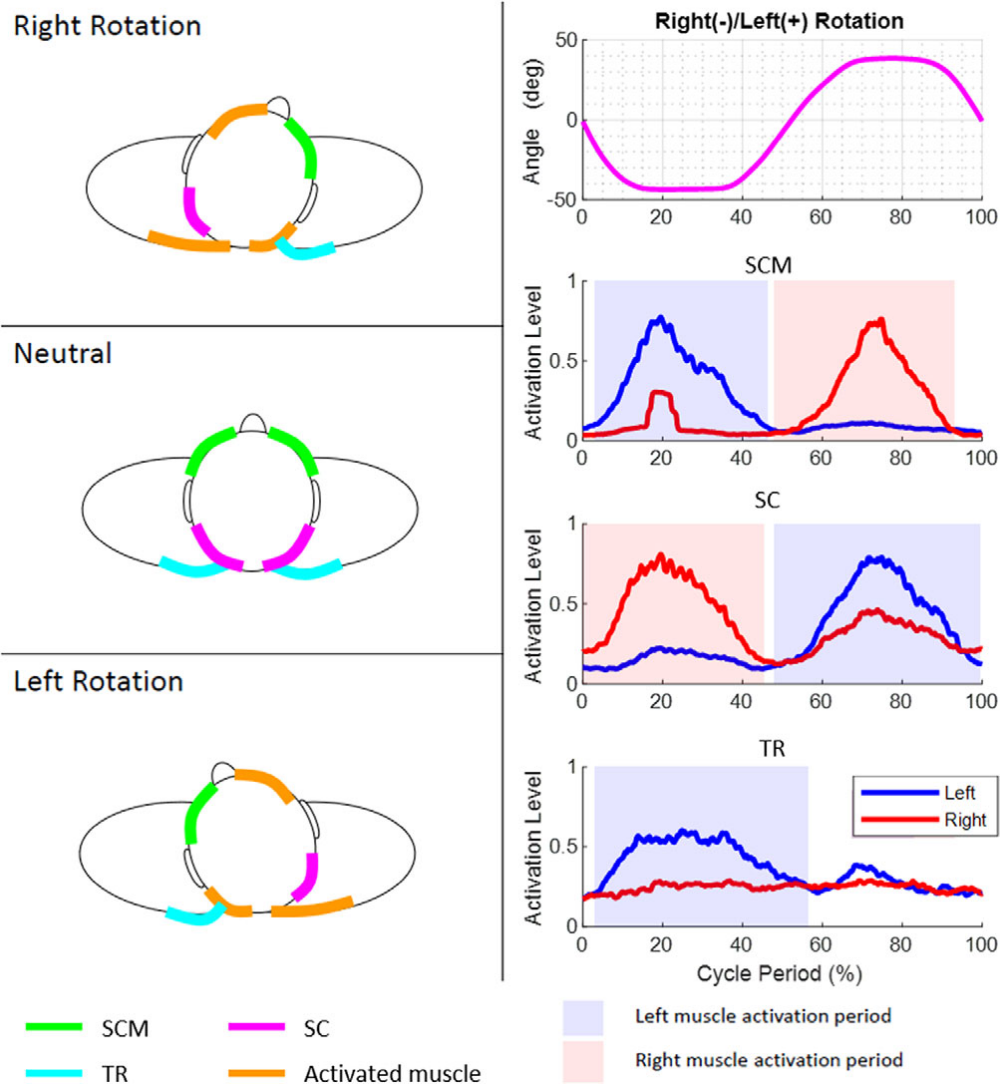
Motion, electromyography (EMG) patterns, and rope activations during axial rotation movement cycle. The axial rotation of the head and neck can be visualized as being caused by a contralateral pair of ropes among sternocleidomastoid (SCM) and splenius capitis (SC) and the ipsilateral trapezius (TR) with SCM.

**Figure 7.**
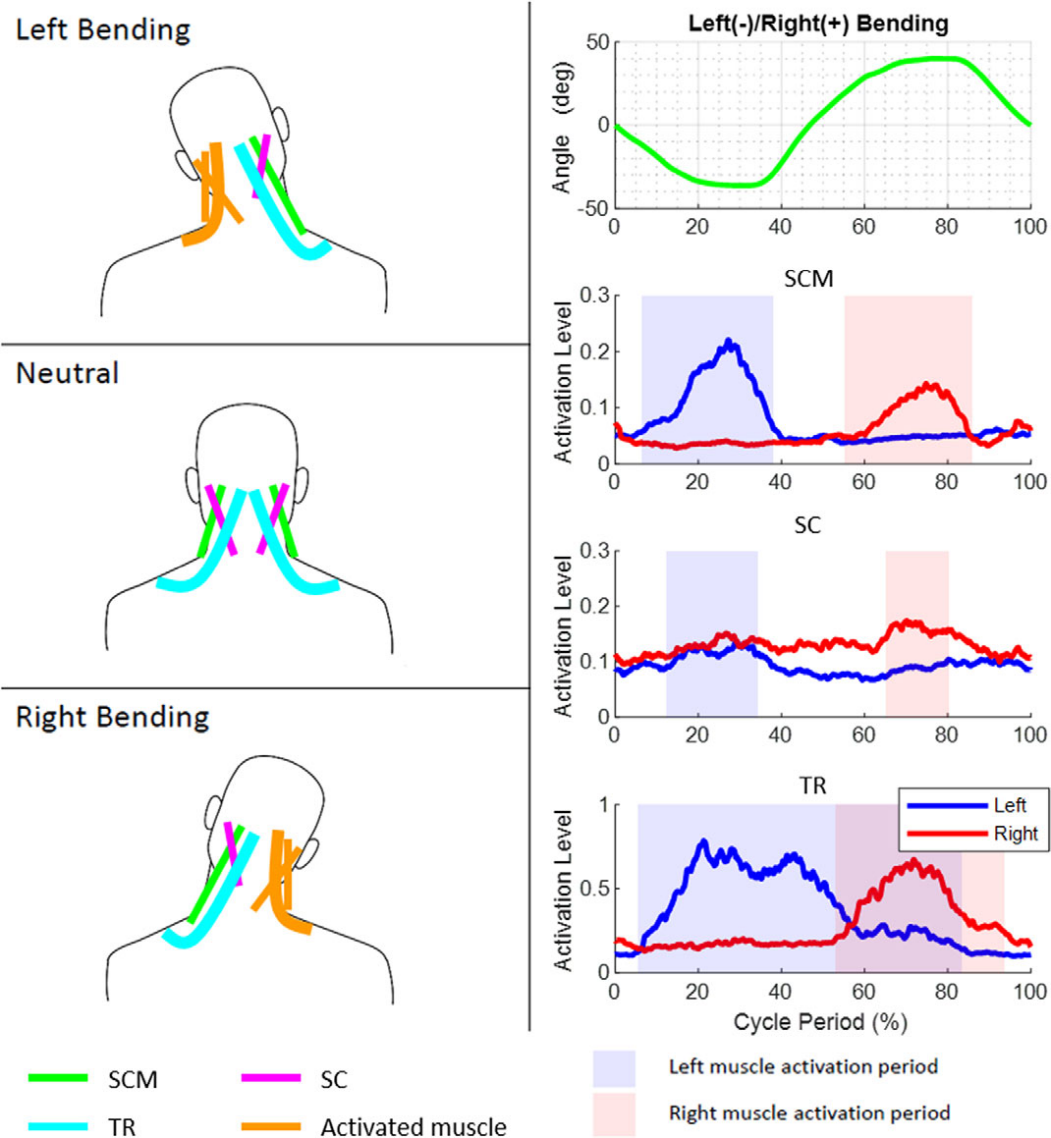
Motion, electromyography (EMG) patterns, and rope activations during bending movement cycle. The lateral bending of the head and neck is caused by three ipsilateral ropes attached between the shoulders and the head.

**Figure 8.**
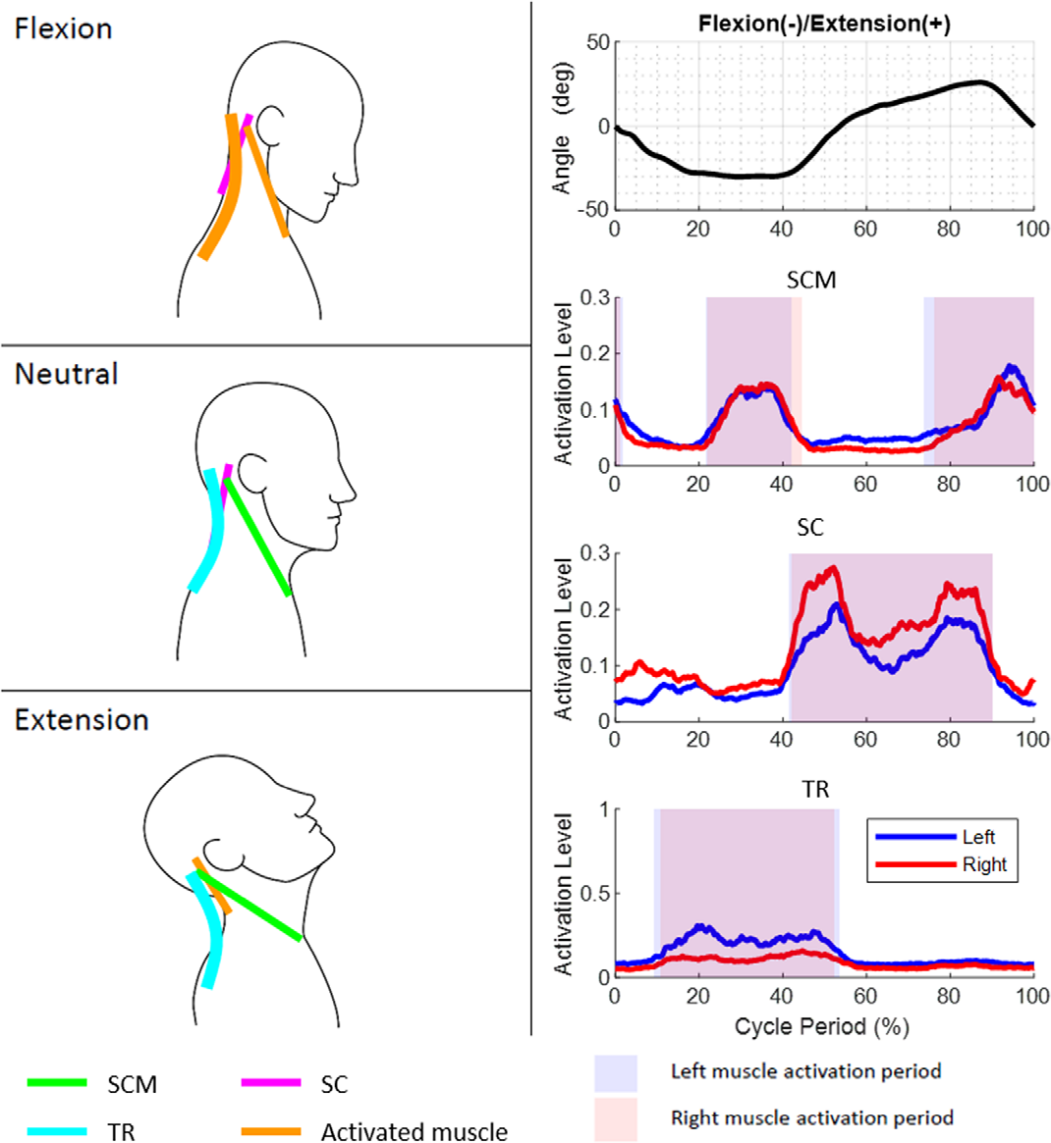
Motion, electromyography (EMG) patterns, and rope activations during flexion–extension movement cycle. The flexion is caused by the two sternocleidomastoid (SCM) while the trapezius (TR) provides stiffness during the motion.

The axial rotation of the head and neck can be visualized as being actuated by a contralateral pair of ropes among SCM and SC and the ipsilateral TR paired with SCM. For example, right axial rotation is achieved by actuation of the left SCM, right SC, and left TR in the rotation cycle. The lateral bending of the head and neck is caused by the three ipsilateral ropes attached between the shoulders and the head. For example, the simultaneous actuation of the left SCM, left SC, and left TR results in left lateral bending. The flexion is caused by the pair of SCM while the TR holds the head to ensure a certain level of stiffness during the motion. Similarly, the extension is caused by the pair of SC. The patterns of these EMGs in relation to the motion peaks for a representative subject before the surgery and the instances within the movement cycle when a particular muscle turns on or off are provided in Figures 6–8.

The group mean time period to complete a cycle was 3.41(0.82) s before surgery and 3.68(1.11) s postoperatively during axial rotation; 4.00(1.00) s before surgery and 3.93(1.04) s postoperatively during lateral bending; and 3.89(1.42) s before surgery and 3.44(0.99) s postoperatively during flexion/extension. No statistically significances were found in all motions.

**Figure 9.**
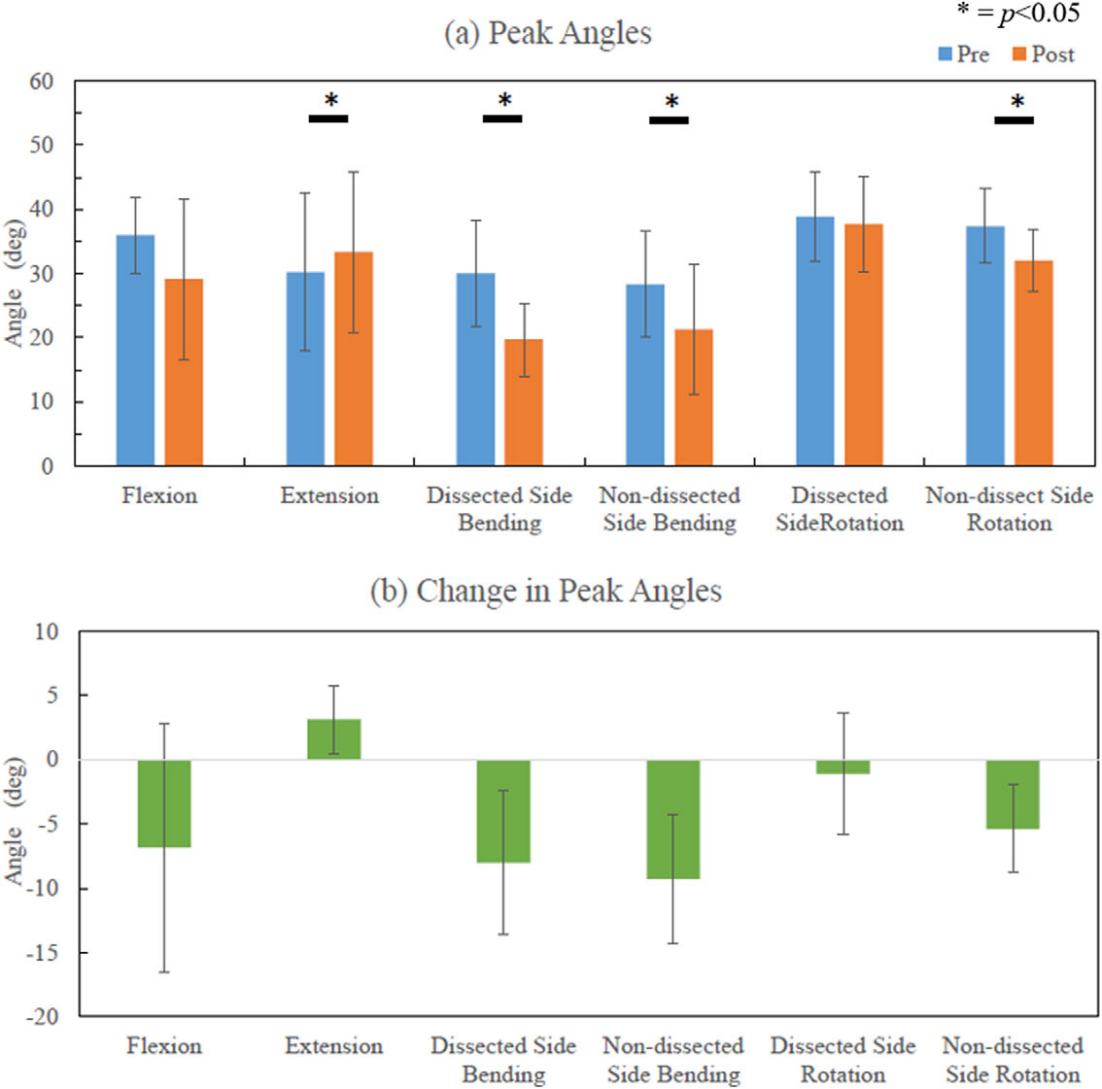
Group data on the peak angles and changes postoperatively. (a) Illustrates peak angles preoperative and postoperative. (b) The change in peak angles.

Figure 9 shows the peak angles for the group during single-plane motions and changes in the peak angles for the group before and after surgery. Except for the extension motion, the peak angles in all other motions decreased after surgery. Lateral bending on both sides showed a significant decrease by 8.03° on dissected side (95% CI, 3.11–12.94, *p* = .016) and 9.29° on the nondissected side (95% CI, 4.88–13.69, *p* = .007). Differences were also observed during axial rotation with the mean decrease of 5.37° (95% CI, 2.34–8.39, *p* = .017) on the contralateral side of ND and during extension with a slight increase of 3.15° (95% CI, 0.81–5.49, *p* = .028).

**Figure 10.**
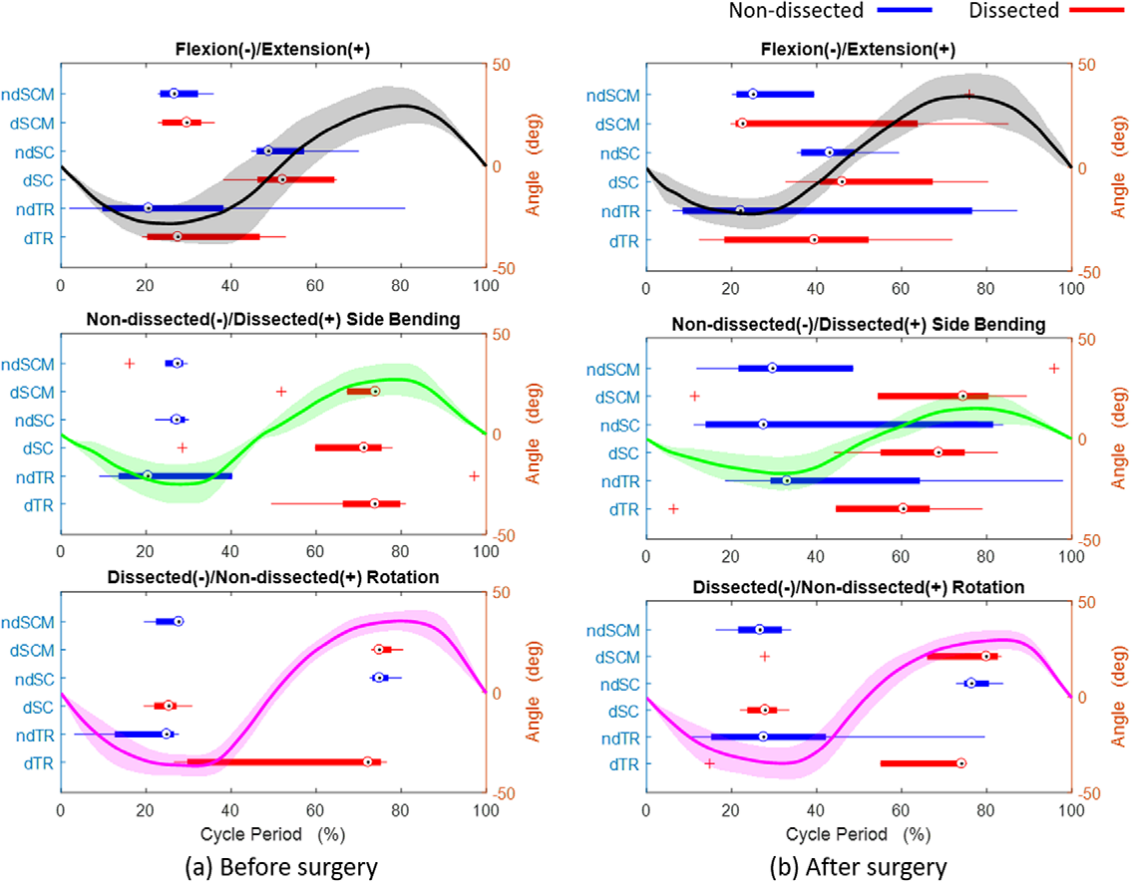
Peak muscle activations during a movement cycle for the group of subjects. (a) Preoperative and (b) 1-month postoperative. Bars represent the temporal spread of the peak activations, circles are median value, and “+” are outliers.

Figure 10 shows the relative timing of the muscle EMG peaks and motion peaks before and 1-month post-surgery. The plots show that the location of EMG peaks had large variation in all motions after surgery among the subjects. However, comparing the peak timing before and after the surgery, significant differences were found in SC on the dissected side during flexion–extension with timing 9% earlier (95% CI, 3–16, *p* = .024) and lateral bending with timing 12% earlier (95% CI, 2–22, *p* = .037). SC on the nondissected side during axial rotation, the timing of the peak was 2% later (95% CI, 0–3, *p* = .042). The timing of maximum extension angle happened 5% earlier and showed a significant difference (95% CI, 1–10, *p* = .037).

## Discussion

In this study, we designed a 6-DOF serial chain neck brace as a measurement tool for the RoM of the head–neck in patients who undergo head–neck surgery. The architecture of the neck brace was selected by understanding the biomechanics during the motion of the head–neck. We observed the neck motion from 10 healthy subjects using a motion capture system and used their data to select and optimize the physical structure of the neck brace. The D–H parameters of the design were chosen so that we could measure full RoM of the head–neck. We optimized the coefficients of the brace model to obtain the characteristic of the device. We validated the performance of the device with two subjects and showed that the RMS errors were within 5°. The device was then used to measure the RoM changes before and after the ND of five patients during their regular clinical visits. The results showed that the brace captured a significant decrease in lateral bending after the surgery. Integrated with the EMG system, we also found that SC muscles activated earlier during the single-plane motions postoperatively.

There are several advantages of using this device as a clinical measurement tool. It is a simple device to use and is wearable by attaching its base to the end of the cervical vertebrae and the end-effector to the top of the head. This can be easily done by an attending clinician. Additionally, this device fits subjects of different body types. Furthermore, using low-cost potentiometers to measure the head angles provides reliable measurement, which may not be achieved by an expensive IMU due to the signal drift over long periods of time. We also have shown in this study that this tool can be easily paired with surface EMG measurement to study the neck movement on the muscular level.

Comprehensive rehabilitation of head and neck cancer patients following ND is a major unmet need (McEwen et al., 2016). Mucosal cancers of the head and neck are the sixth most common forms of cancer in the United States affecting approximately 65,000 patients per year (Howlader et al., 2017a). Rising rates of skin cancers and human papilloma virus associated cancers of the tonsil and tongue are resulting in a large population of patients living with treatment-related morbidity considering the need for cervical lymphadenectomy along with primary resection. Early age at presentation and high cure rates of such cancers translate to more patient-years of life lived with decreased quality of life after surgical therapy. When questioned, patients have consistently identified “guided exercise” and “physical therapy” as two unmet needs (McEwen et al., 2016). While ND offers increased diagnostic and therapeutic advantage, it is not uncommon for there to be iatrogenic injuries to vital neurovascular structures, including the accessory nerve (Köybasioglu et al., 2000). These have also been identified by patients as needs most frequently overlooked by physicians, in particular with the need for increased psychosocial support. An easy-to-use, precise, and reproducible screening tool is needed to identify patients who suffer from ND associated movement disorders. Such a tool can be used to identify patients who are in need for targeted interventions, to develop evidence based rehabilitative programs, and to measure accurately the response of treatments.

Denervation injury and associated movement disorders are often not apparent until the patients have left the hospital. This delay in presentation leads to under detection of ND related movement disorders as patients fail to fully report these symptoms and physicians overlook these issues given the time constraints of outpatient cancer visits. The development of a screening tool can help identify patients suffering from decreased quality-of-life and those who should be targeted for physical therapy interventions.

Most studies have focused on movement disorders of the shoulders but investigations of movement disorders in the neck are currently lacking. Movement restrictions and compensatory patterns of neck muscles have not been fully described. Previous studies have compared the dysfunction and quality of life differences between nerve-sacrificing and nerve-sparing ND. Leipzig et al. (1983) reported that patients had less dysfunction with nerve preserving selective lymphadenectomy compared to those who underwent nerve-sacrificing radical ND. Cheng et al. (2000) showed patients with selective ND had less nerve damage and therefore less shoulder disability. Eickmeyer et al. (2014) demonstrated that patients who had nerve-sacrificing ND reported poorer quality of life score and poorer shoulder functioning after 5 years.

In the group data, the peak flexion angle, lateral bending on both sides, and nondissection side rotation decreased (Figure 9). The rotation on the nondissected side is controlled by SCM and TR on the dissected side and thus appeared diminished postoperatively. The mechanical stiffness of the brace may influence the stiffness of the head and neck, however, this influence is there both before and after surgery. After surgery, subjects tended to have their own strategy to perform the movement, which was captured in the variability of the timing of the peak muscle activations after the surgery (Figure 10). However, significant differences were found in the peak timing of SC on the dissected side during lateral bending and flexion/extension, which may relate to the significant differences found in RoMs. Thus, the peak timing on SC could influence maximum RoM of the neck. Since we have small sample size and different people may have distinct strategies, we did not find the correlation between the RoM and EMG.

One of the limitations of this device is that the errors progressively add due to the serial chain structure of the mechanism. However, a 5° error is acceptable when the user is performing a large head orientation, in excess of 100°. Motion capture systems require a long set up time in the clinic, which make these impractical during routine visits. This study shows that the brace can be used to measure relative angle changes before and after the surgery. The accuracy and repeatability of the angle measurements can be further improved in the future.

## Conclusions

In this study, we presented a neck brace based on a serial mechanism. The goal was to design a low-cost, wearable, and easy-to-use device to measure the full range of motion of the head–neck. The brace was designed with head–neck movement data from 10 healthy young subjects to determine the physical structure of this device. The system was then used to study preoperative and postoperative ND performance.

The study suggests that restricted range of neck mobility occurs in patients after ND, even in the era of selective lymphadenectomy and nerve-sparing procedures. The portability, accuracy, and ease with which the data were collected suggest that the neck brace may serve as surgical procedure evaluation tool in a head and neck surgery or rehabilitative medicine practice. The first is as a screening tool that can accurately detect the presence of mobility restriction in patients undergoing surgery. The second is a method of identifying the muscle group most in need of therapeutic intervention. Finally, the brace can be used as a method of objectively quantifying the return of mobility as patients’ progress through postoperative physical therapy.

## Data Availability

Data can be made available to interested researchers upon request by email to the corresponding author.
