# A novel neck brace to characterize neck mobility impairments following neck dissection in head and neck cancer patients – ADDENDUM

**DOI:** 10.1017/wtc.2024.3

**Published:** 2024-02-16

**Authors:** Biing-Chwen Chang, Haohan Zhang, Sallie Long, Adetokunbo Obayemi, Scott H. Troob, Sunil K. Agrawal

The authors regret the omission of the Ethical Standards and Consent for Publication statements in the above article. These statements are provided below:

**Ethical standards**

The authors assert that all procedures contributing to this work comply with the ethical standards of the relevant national and institutional committees on human experimentation and with the Helsinki Declaration of 1975, as revised in 2008. The study was approved by the Institutional Review Board of Columbia University under the approval number #AAAQ7702. All participants gave written informed consent for experiment participation and protected health information access after a complete explanation of this study.

**Consent for publication**

All participants gave written informed consent for publication after a complete explanation of this study.

The original article has been updated.

